# Supplementary material for: Unveiling excitonic insulator signatures in Ta[image]NiSe[image] through structural and orbital probes
Source: Sci Rep. 2025 Oct 7;15:35019. doi: 10.1038/s41598-025-20909-7 (PMC12504696; doi:10.1038/s41598-025-20909-7)
Supplement: Supplementary file 1 — Supplementary Information. [file 41598_2025_20909_MOESM1_ESM.pdf]

Unveiling Excitonic Insulator Signatures in Ta_2NiSe_5 through Structural and Orbital Probes

Nour Maraytta,[1,*] Peter Nagel,[1,2] Fatemeh Ghorbani,[1] Amir Ghiami,[1,2] Santanu Pakhira,[1] Mai Ye,[1] Björn Wehinger,[3] Federico Abbruciati,[3] Gaston Garbarino,[3] Matthieu Le Tacon,[1] Stefan Schuppler,[1,2] Amir-Abbas Haghighirad,[1] and Michael Merz[1,2,*]

[1]*Institute for Quantum Materials and Technologies, Karlsruhe Institute of Technology, Kaiserstr. 12, 76131 Karlsruhe, Germany*

[2]*Karlsruhe Nano Micro Facility (KNMFi), Karlsruhe Institute of Technology, Kaiserstr. 12, 76131 Karlsruhe, Germany*

[3]*ESRF, The European Synchrotron, 71, avenue des Martyrs, CS 40220 F-38043 Grenoble Cedex 9, France*

[*]*Corresponding authors: nour.maraytta@kit.edu, michael.merz@kit.edu*

(Dated: September 21, 2025)

CONTENTS

1. Sample Fabrication and Characterization 3

2. XRD: Structure Determination and Refinement 4

3. Laue Diffraction 11

4. Near Edge X-ray Absorption Fine Structure (NEXAFS) 12

References 14

The Supplemental Material contains further information on the sample preparation and characterization, temperature-dependent single-crystal x-ray diffraction (SC-XRD), Laue diffraction, and near-edge x-ray absorption fine structure (NEXAFS) spectra.

1. SAMPLE FABRICATION AND CHARACTERIZATION

High-quality single crystals of $Ta_2(Ni_{1-x}Co_x)Se_5$ were grown by chemical vapor transport. Starting material in powder form was prepared by solid-state reaction. High-purity (at least 99.99%) starting materials of Ta:Ni:Co:Se with a nominal ratio of 2:0.9:0.1:5 were mixed, weighed and subsequently reacted in a sealed fused silica ampoule at 700 °C for 7 days. The obtained product was ground in an agate mortar, mixed with $\sim$ 100 mg iodine as a transport agent, and sealed in an evacuated quartz ampoule. The quartz tube was placed in a horizontal two-zone tube furnace. The temperatures of the zones were kept at 900 °C (i.e., for the source materials) and 825 °C (for the growth region), respectively. The duration of the transport was 12 days. Shiny single-crystals of typical dimensions 10 mm $\times$ 2 mm $\times$ 0.2 mm (see Fig S1) were formed in the cooler zone of the ampoule. Ta_2NiSe_5 and Ta_2NiS_5 single crystals were grown in the corresponding way as described in [1] and [2].

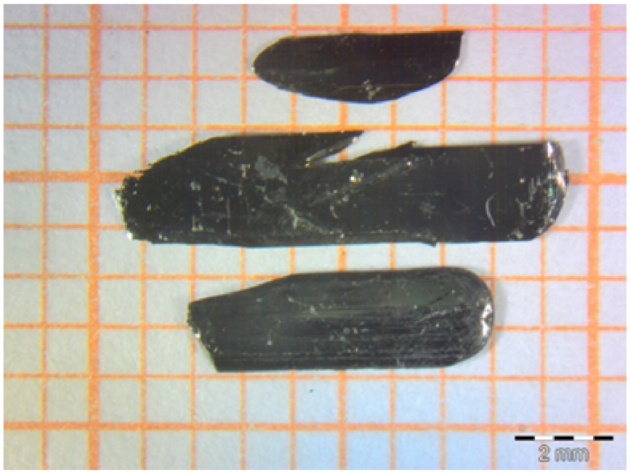

FIG. S1. $Ta_2(Ni_{0.925}Co_{0.075})Se_5$ single crystals grown by chemical vapor transport.

The chemical composition of a $Ta_2(Ni_{1-x}Co_x)Se_5$ single crystal was determined using energy dispersive x-ray spectroscopy (EDS) in a COXEM EM-30plus electron microscope equipped with an Oxford Silicon-Drift-Detector (SDD) and AZtecLiveLite-software package. EDS revealed a quantitative elemental composition of a $Ta_2(Ni_{1-x}Co_x)Se_5$ crystal with a

cobalt substitution level of x = 0.075. The EDS spectrum of the quantitative elemental mapping is shown in Fig. S2. The detailed sample characterization of Ta_2NiSe_5 and Ta_2NiS_5 single crystals is provided in [1] and [2], respectively.

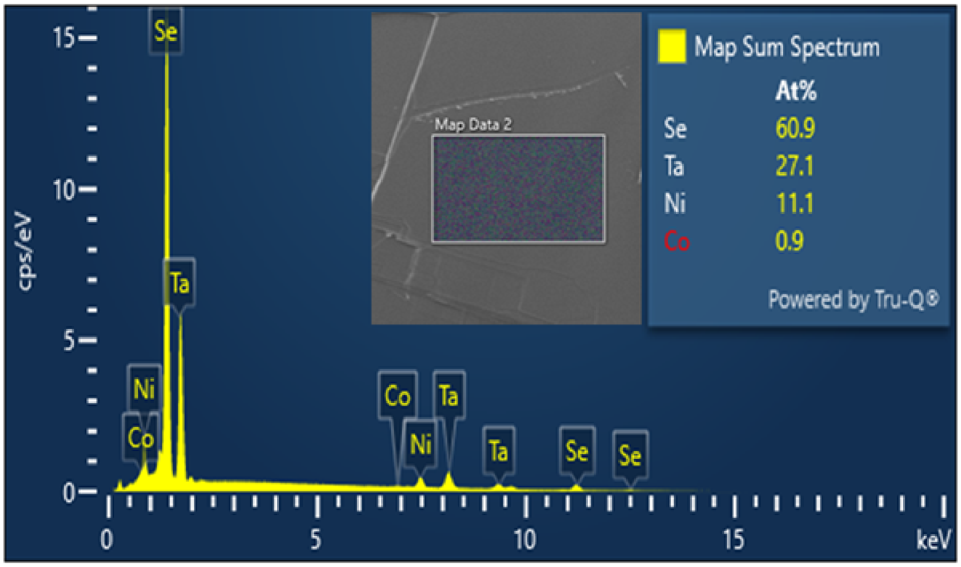

FIG. S2. Electron probe microanalysis $Ta_2(Ni_{1-x}Co_x)Se_5$ (x = 0.075). EDX spectrum of the surface of a $Ta_2(Ni_{1-x}Co_x)Se_5$ single crystal – inset: elemental mapping area and the quantitative analysis of a $Ta_2(Ni_{1-x}Co_x)Se_5$ sample.

2. XRD: STRUCTURE DETERMINATION AND REFINEMENT

Temperature-dependent SC-XRD data on Ta_2NiSe_5, $Ta_2Ni_{0.93}Co_{0.07}Se_5$, and Ta_2NiS_5 were collected between 360 K and 80 K using our in-house high-flux, high resolution, rotating anode Rigaku Synergy-DW (Mo/Ag) diffractometer with Mo K_α radiation. Data evaluation was performed using the latest version of the CrysAlisPro software package [3]. The crystal structures of the compounds were refined using JANA2006 [4]. For each measured temperature, the unit cell and space group were determined, atoms were localized within the unit cell using random phases and the structure was completed and solved using difference Fourier analysis.

Table S1 shows the crystallographic results of Ta_2NiSe_5, $Ta_2Ni_{0.93}Co_{0.07}Se_5$, and Ta_2NiS_5 in the high-temperature phase, where the structure was refined in the orthorhombic space group $Cmcm$ for which $\alpha = \beta = \gamma = 90°$. Table S2 shows the results in the low temperature phase (80 K) where the structure was refined in the monoclinic SG $C2/c$ for which $\beta \neq 90°$.

The lattice parameters a, b, and c, the volume V of the unit cell, and the relative atomic positions (x, y, and z) are shown in the Tables. The ADPs were refined anisotropically but due to space limitations only the equivalent atomic displacement parameters (U_{eq}) are presented. Ta-Ni and Ta-Se3/S3 in the high-temperature phase (Ta-Nia/b and Ta-Se3a/Se3b and Ta-S3 in the low-temperature phase) represent the bond lengths between the corresponding atoms. Errors shown are statistical errors from the refinement. The structural refinements converged well for all investigated temperatures, exhibiting excellent reliability factors (see wR_2, R_1, and GOF values in Table S1 and Table S2).

TABLE S1. Crystallographic results of Ta_2NiSe_5, $Ta_2Ni_{0.93}Co_{0.07}Se_5$, and Ta_2NiS_5 in the high-temperature phase determined from single crystal x-ray diffraction. The high-temperature structure was refined in the orthorhombic space group (SG) $Cmcm$ for which $\alpha = \beta = \gamma = 90°$. The lattice parameters a, b, and c are shown together with the volume V of the unit cell. Ta, Se2/S2, and Se3/S3 occupy a $8f$ Wyckoff position (WP), Ni/Co and Se1/S1 a $4c$ position. Relative atomic positions (x, y, and z) are shown in the Table. U_{eq} denotes the equivalent atomic displacement parameters (ADP). The ADPs were refined anisotropically but due to space limitations only the U_{eq} are listed in the Table. Ta-Ni (Ta-Se3/S3) represents the bond lengths between the corresponding atoms. Errors shown are statistical errors from the refinement.

		$\mathbf{Ta_2NiSe_5}$ (350 K)	$\mathbf{Ta_2Ni_{0.93}Co_{0.07}Se_5}$ (350 K)	$\mathbf{Ta_2NiS_5}$ (360 K)
	a (Å)	3.5006(1)	3.4919(1)	3.4184(1)
	b (Å)	12.8542(5)	12.8244(3)	12.1682(3)
	c (Å)	15.6567(8)	15.6515(3)	15.108(4)
	V (Å^3)	704.511035	700.897977	628.428944
**Ta**	x	0	0	0
	y	0.22120(2)	0.22118(1)	0.22070(1)
	z	0.61027(1)	0.61027(1)	0.60864(1)
	U_{eq}	0.01099(6)	0.01152(3)	0.01108(3)
**Ni/Co**	x	0	0	0
	y	0.29903(9)	0.29899(6)	0.30320(5)
	z	$\frac{1}{4}$	$\frac{1}{4}$	$\frac{1}{4}$
	U_{eq}	0.0120(3)	0.0129(1)	0.0125(1)
**Se1/S1**	x	0	0	0
	y	0.67308(7)	0.67299(5)	0.68085(8)
	z	$\frac{1}{4}$	$\frac{1}{4}$	$\frac{1}{4}$
	U_{eq}	0.01130(18)	0.01186(10)	0.0117(2)
**Se2/S2**	x	0	0	0
	y	0.14571(5)	0.14581(3)	0.14862(5)
	z	0.04928(4)	0.04925(2)	0.05031(4)
	U_{eq}	0.01033(13)	0.01093(7)	0.01086(14)
**Se3/S3**	x	0	0	0
	y	0.41948(5)	0.41970(3)	0.41708(6)
	z	0.13781(4)	0.13788(3)	0.13481(5)
	U_{eq}	0.01207(14)	0.01236(8)	0.01252(14)
	Ta–Ni	2.8138(3)	2.8103(2)	2.75081(2)
	Ta-Se3/S3	2.5533(5)	2.5493(3)	2.4265(5)
GOF		1.48	1.45	1.17
wR_2 (%)		6.27	8.55	4.27
R_1(%)		3.52	7.51	2.64

TABLE S2. Crystallographic results of Ta_2NiSe_5, $Ta_2Ni_{0.93}Co_{0.07}Se_5$, and Ta_2NiS_5 in the low-temperature phase (80 K) determined from single crystal x-ray diffraction. The low-temperature structure was refined in the monoclinic SG $C2/c$ for which $\alpha = \gamma = 90°$. The lattice parameters a, b, c, and β are shown together with the volume V of the unit cell. Ta, Se2/S2, and Se3/S3 occupy a $8f$ Wyckoff position (WP), Ni/Co and Se1/S1 a $4e$ position. Relative atomic positions (x, y, and z) are shown in the table. U_{eq} denotes the equivalent atomic displacement parameters (ADP). The ADPs were refined anisotropically but due to space limitations only the U_{eq} are listed in the table. Ta-Nia/b [Ta-Se3/S3(a/b)] represents the bond lengths between the corresponding atoms. Errors shown are statistical errors from the refinement.

		Ta_2NiSe_5	$Ta_2Ni_{0.93}Co_{0.07}Se_5$	Ta_2NiS_5
	a (Å)	3.4866(1)	3.4805(2)	3.4085(0)
	b (Å)	12.8001(3)	12.7995(5)	12.1210(2)
	c (Å)	15.6288(4)	15.6165(7)	15.0791(3)
	β (°)	90.614(2)	90.475(4)	90
	V (Å^3)	697.455008	695.700122	622.984391
**Ta**	x	0.48792(5)	0.48814(6)	0
	y	0.22132(2)	0.221335(10)	0.221014(11)
	z	0.11061(1)	0.110574(9)	0.108899(10)
	U_{eq}	0.00400(6)	0.00460(2)	0.00500(4)
**Ni/Co**	x	0	0	0
	y	0.20111(10)	0.20120(5)	0.19744(6)
	z	$\frac{1}{4}$	$\frac{1}{4}$	$\frac{1}{4}$
	U_{eq}	0.0046(2)	0.00511(11)	0.00572(15)
**Se1/S1**	x	0	0	0
	y	0.82736(7)	0.82750(4)	0.82019(11)
	z	$\frac{1}{4}$	$\frac{1}{4}$	$\frac{1}{4}$
	U_{eq}	0.00416(18)	0.00491(9)	0.0059(3)
**Se2/S2**	x	0.00857(13)	0.0083(2)	0
	y	0.35450(5)	0.35450(2)	0.35177(7)
	z	0.04895(4)	0.04897(2)	0.049990(6)
	U_{eq}	0.00403(13)	0.00473(6)	0.00563(19)
**Se3/S3**	x	0.00811(13)	0.0067(2)	0
	y	0.08012(5)	0.07991(3)	0.08266(8)
	z	0.13805(4)	0.13805(2)	0.13497(7)
	U_{eq}	0.00482(14)	0.00508(6)	0.00597(19)
	Ta–Nia	2.7904(3)	2.7848(3)	2.74101(15)
	Ta–Nib	2.8137(3)	2.8137(3)	-
	Ta-Se3a/S3	2.5029(6)	2.5062(6)	2.4231(7)
	Ta-Se3b	2.5930(6)	2.5894(6)	-
GOF		1.59	1.64	1.39
wR_2 (%)		7.25	8.11	5.29
R_1(%)		4.53	7.92	3.41

Fig. S3, Fig. S4, and Fig. S5 represent precession images for the reciprocal planes (h 0 l), (h 1 l), and (h 3 l) respectively, reconstructed from single-crystal XRD data collected at 360 and 80 K for Ta_2NiSe_5 (left), 280 and 80 K for $Ta_2Ni_{0.93}Co_{0.07}Se_5$ (middle), and 360 and 80 K for Ta_2NiS_5 (right). For Ta_2NiSe_5 and $Ta_2Ni_{0.93}Co_{0.07}Se_5$, a splitting of the reflections below the orthorhombic-to-monoclinic phase transition is obvious. For Ta_2NiS_5, in the entire reciprocal lattice, and particularly in the (h k l) layers with k = 0, 1, 2, 3, ... no evidence of reflection splitting is observed, which clearly indicates that the compound does not undergo any phase transition down to 80 K.

To ensure that no phase transition occurs below 80 K, additional synchrotron XRD measurements were performed down to 2 K at the ID15B beamline of the European Synchrotron Radiation Facility (ESRF) [5]. None of the accessible reflections in the (h k l) planes with k = 0, 1, 2, 3, ... showed any signs of a splitting or a broadening expected in the case of a twinned crystal below the phase transition—as a representative example see Fig. S4 for precession images of the reciprocal (h 1 l) (left), and (h 3 l) (right) planes. Thus, we can rule out any structural phase transition in our samples down to 2 K.

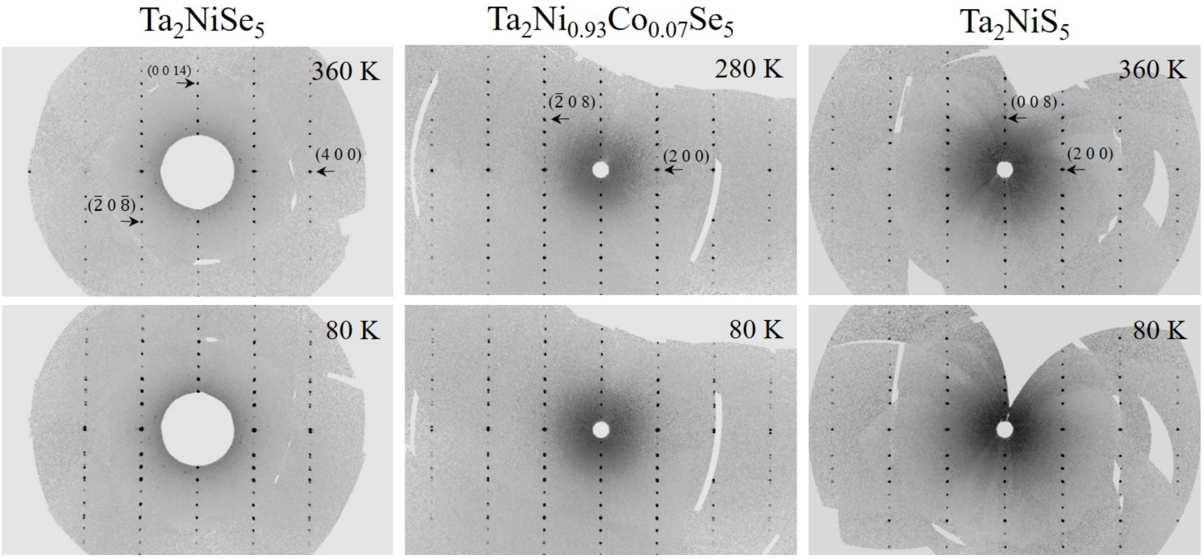

FIG. S3. Precession images for a reciprocal (h 0 l) plane of Ta_2NiSe_5 (left), $Ta_2Ni_{0.93}Co_{0.07}Se_5$ (middle), and Ta_2NiS_5 (right) reconstructed from single-crystal XRD data collected on our in-house diffractometer.

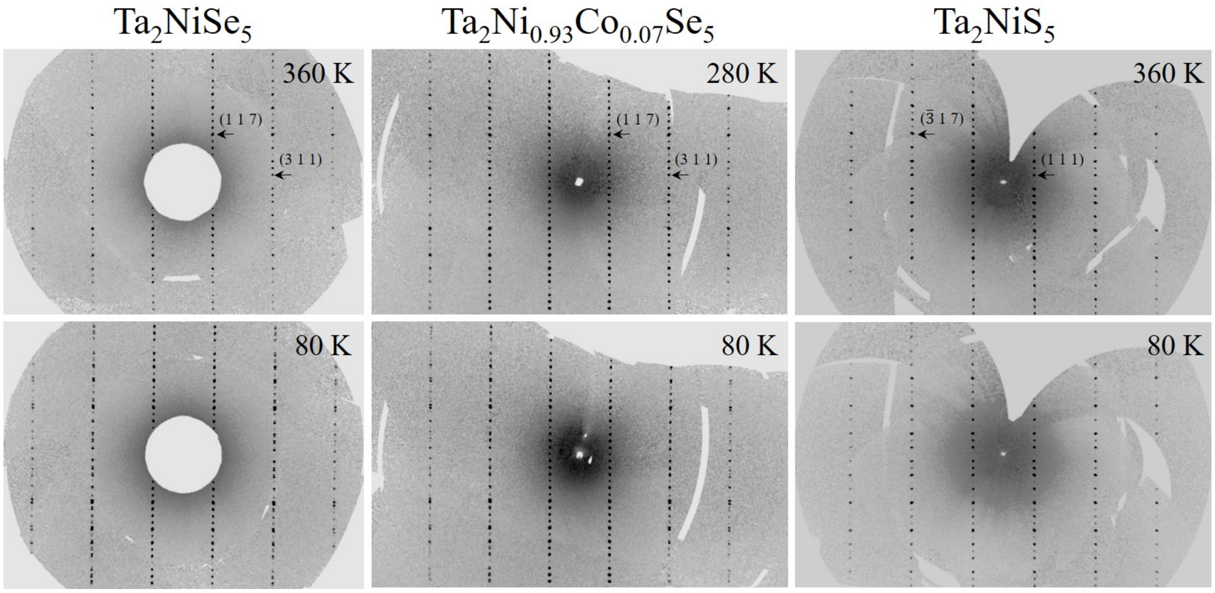

FIG. S4. Precession images for a reciprocal (h 1 l) plane of Ta_2NiSe_5 (left), $Ta_2Ni_{0.93}Co_{0.07}Se_5$ (middle), and Ta_2NiS_5 (right) reconstructed from single-crystal XRD data collected on our in-house diffractometer.

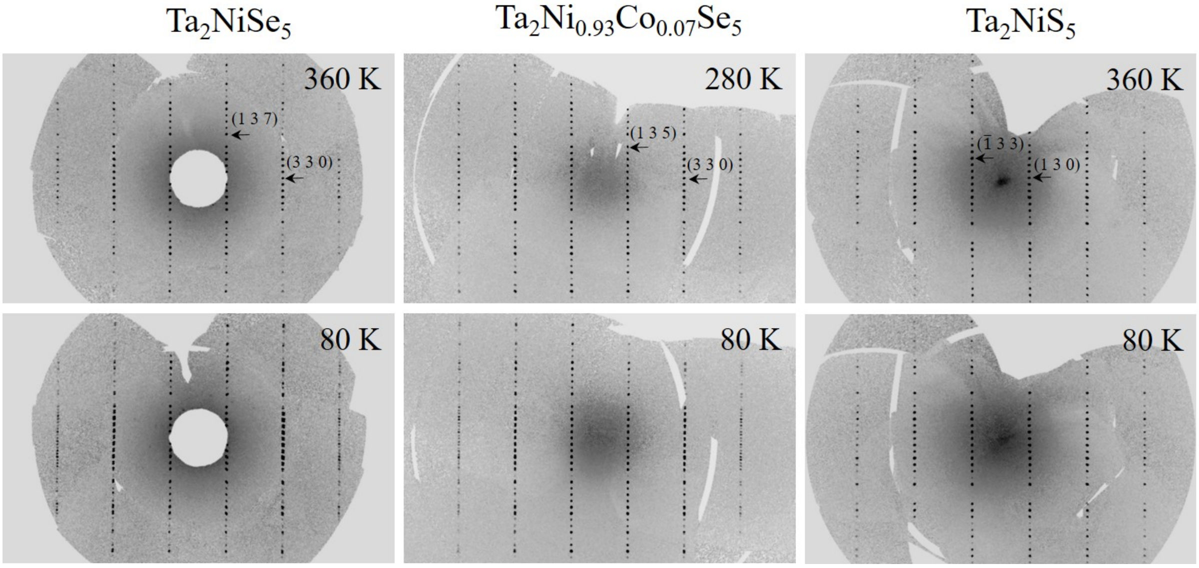

FIG. S5. Precession images for a reciprocal (h 3 l) plane of Ta_2NiSe_5 (left), $Ta_2Ni_{0.93}Co_{0.07}Se_5$ (middle), and Ta_2NiS_5 (right) reconstructed from single-crystal XRD data collected on our in-house diffractometer.

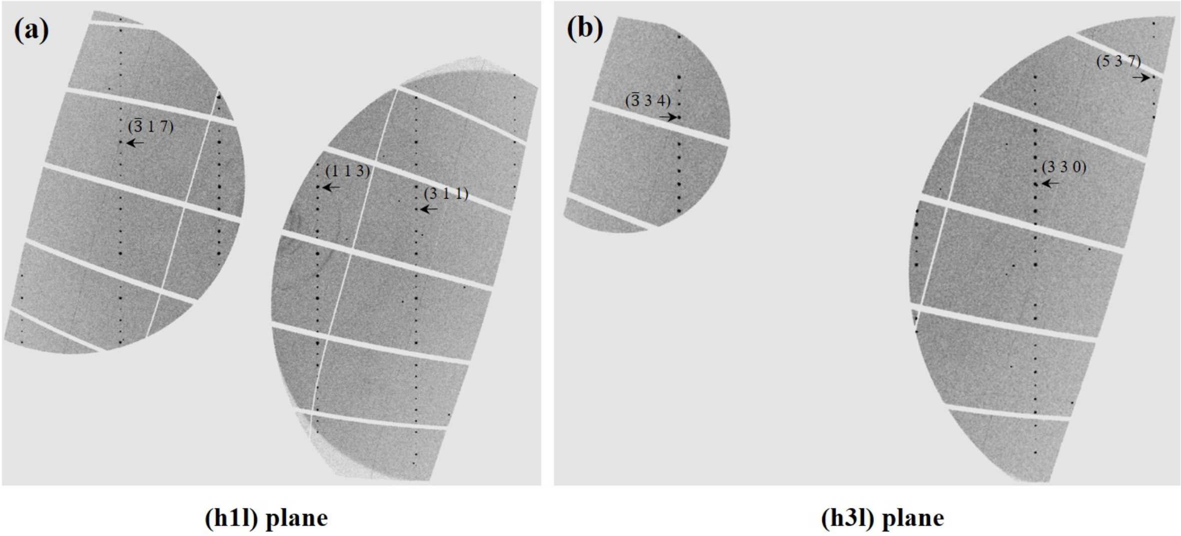

FIG. S6. Precession images for reciprocal (a) (h 1 l) and (b) (h 3 l) planes of Ta_2NiS_5 reconstructed from single-crystal XRD data collected at 2 K on the diffractometer of the ID 15B beamline at the ESRF.

3. LAUE DIFFRACTION

The orientations of the individual samples used in our NEXAFS studies were precisely determined using a fully motorized Photonic Science Laue diffraction system. Laue diffraction images were simulated based on the lattice parameters derived from our single-crystal XRD measurements, as shown in Fig. S7. From Laue diffraction images together with the calculated simulation, it was found that the crystallographic b direction is perpendicular to the plane of the image, the a direction is horizontal, and the c direction is vertical. For both a Ta_2NiSe_5 as well as a $Ta_2Ni_{0.93}Co_{0.07}Se_5$ specimen, representative examples of the respective sample orientation are illustrated in Fig. S7.

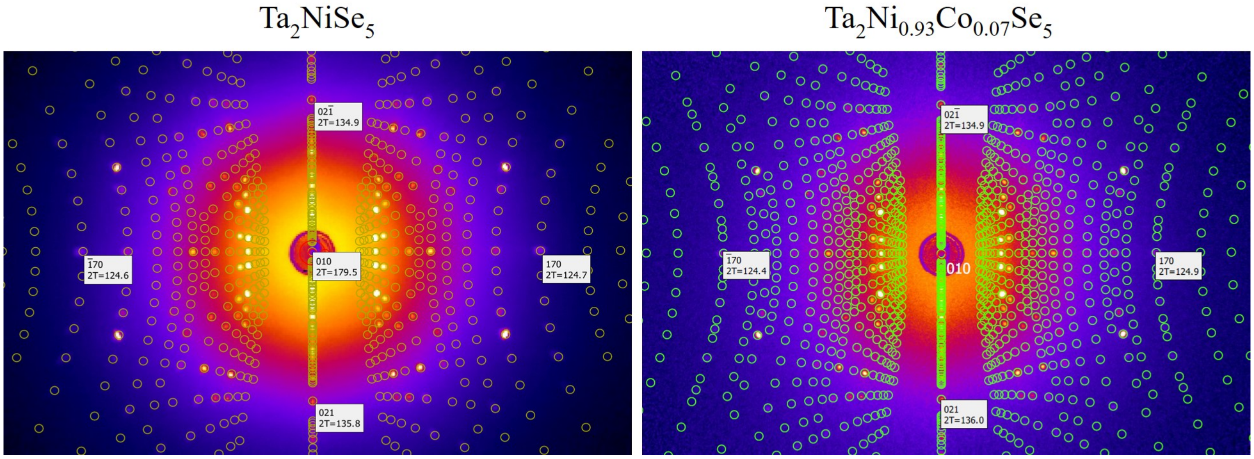

FIG. S7. Photonic Science Laue diffraction system image together with the calculated simulation based on the lattice parameters derived from our single-crystal XRD measurements on Ta_2NiSe_5 (left) and $Ta_2Ni_{0.93}Co_{0.07}Se_5$ (right). The crystallographic b direction is perpendicular to the plane of the image, the a direction is horizontal, and the c direction is vertical.

4. NEAR EDGE X-RAY ABSORPTION FINE STRUCTURE (NEXAFS)

Temperature-dependent NEXAFS measurements were performed at IQMT's WERA beamline at the KIT Light Source in Karlsruhe, Germany. NEXAFS data were collected for Ta_2NiSe_5, $Ta_2Ni_{0.93}Co_{0.07}Se_5$, and Ta_2NiS_5 at the $L_{2,3}$ edges of Ni with the polarization of the beam parallel to the a,b, and c directions, respectively. As an example, Fig. S8 presents the complete NEXAFS spectrum of a $Ta_2Ni_{0.93}Co_{0.07}Se_5$ sample measured at the Ni $L_{2,3}$ edges at 390 K with the polarization of the beam parallel to the a direction.

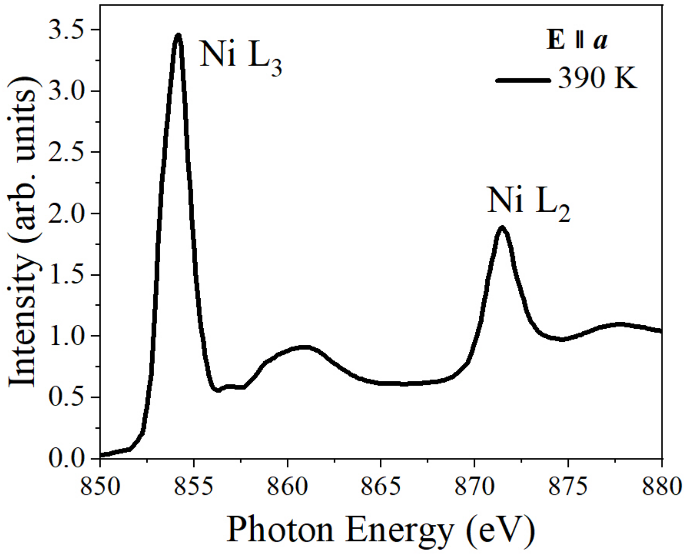

FIG. S8. Complete NEXAFS spectrum of $Ta_2Ni_{0.93}Co_{0.07}Se_5$ at the Ni $L_{2,3}$ edges with the polarization of the beam parallel to the a direction. Shown is the spectrum taken at 390 K.

Fig. S9 shows NEXAFS spectra of Ta_2NiSe_5, $Ta_2Ni_{0.93}Co_{0.07}Se_5$, and Ta_2NiS_5 recorded at 390 K, 290 K, 150 K, and 30 K with the polarization of the beam parallel to the a direction. A significant spectral shift is observed between the curves above and below the transition in Ta_2NiSe_5 and $Ta_2Ni_{0.93}Co_{0.07}Se_5$, which is totally absent in the Ta_2NiS_5 spectra. To interpret the data in Fig. S9, it is helpful to first examine the order parameter β shown in Fig. 1(a) of the manuscript. For Ta_2NiSe_5, we observe a second-order structural transition at 327 K, with β changing continuously down to 80 K without a clear flattening. Consistently, Fig. S9 (left) shows that the additional gap gradually increases below 327 K as the temperature decreases to the lowest measured temperature. To analyze $Ta_2Ni_{0.93}Co_{0.07}Se_5$, Fig. 1(b) indicates a phase transition around 265 K. It is also clear that the order parameter quickly

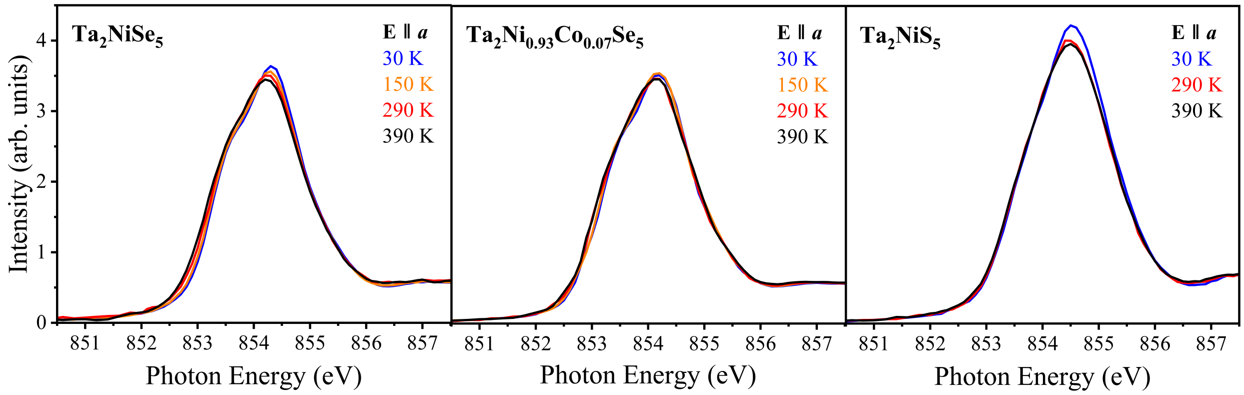

FIG. S9. NEXAFS spectra of (left) Ta_2NiSe_5, (middle) $Ta_2Ni_{0.93}Co_{0.07}Se_5$ and (right) Ta_2NiS_5 taken at 390 K, 290 K, 150 K and 30 K with the polarization of the beam parallel to the a direction. A significant spectral shift is observed in the curves above and below the transition in Ta_2NiSe_5, and $Ta_2Ni_{0.93}Co_{0.07}Se_5$, which is totally absent in the Ta_2NiS_5 spectra.

flattens below approximately 230 K. Above this transition, NEXAFS measurements [Fig. S9 (middle)] were taken at 390 K and 290 K, while below the transition, data were collected at 150 K and 30 K. Consistent with this, the two temperatures above and below the phase transition tend to pair up, with a reduced but still clearly observable gap between high- and low-T phase data. In contrast, for Ta_2NiS_5 [Fig. S9 (right)], no phase transition occurs, and as a result, no additional gap develops. This suggests that excitonic insulator behavior is absent in this system, whereas it appears to be present in Ta_2NiSe_5 and $Ta_2Ni_{0.93}Co_{0.07}Se_5$.

Furthermore, as explained in the manuscript, the small intensity changes, along with the direction-dependent redistribution of spectral weight observed at the phase transition (see Fig. 4 of the manuscript and the related discussion), suggest a change in the orbital character from in-plane to out-of-plane. A change in the valence or spin state would produce more significant modifications in the multiplet structures and can—consistent with our structural data—be ruled out.

[1] K. Katsumi, A. Alekhinand, S. M. Souliou, M. Merz, A. A. Haghighirad, M. L. Tacon, S. Houver, M. Cazayous, A. Sacuto, and Y. Gallais, Disentangling lattice and electronic instabilities in the excitonic insulator candidate Ta_2NiSe_5 by nonequilibrium spectroscopy, Phys. Rev. Lett. **130**, 106904 (2023).

[2] M. Ye and T. Lacmann and M. Frachet and I. Vinograd and G. Garbarino and N. Maraytta and M. Merz and R. Heid and A.-A. Haghighirad and M. Le Tacon, Anomalous phonon grüneisen parameters in the semiconductor Ta_2NiS_5, Phys. Rev. B **110**, 035120 (2024).

[3] Rigaku Oxford Diffraction Ltd, CrysAlisPro software system, version 1.171.44, Rigaku Corporation, Wroclaw, Poland, Rigaku Oxford Diffraction, Yarnton, Oxfordshire, E 2015 CrysAlisPro.

[4] V. Petříček and M. Dušek and L. Palatinus, Crystallographic computing system jana2006: General features, Zeitschrift für Kristallographie - Crystalline Materials **229**, 345 (2014).

[5] G. Garbarino, M. E. Hanfland, S. Gallego-Parra, A. Rosa, M. Mezouar, D. Duran, K. Martel, E. Papillon, T. Roth, P. Got, and J. Jacobs, Extreme conditions x-ray diffraction and imaging beamline id15b on the esrf extremely brilliant source, High Press. Res. **44**, 199 (2024).
